# Supplementary material for: Ethnicity and trends in pediatric specialty care clinic attendance at an academic medical center in the rural southeastern US
Source: PLOS Glob Public Health. 2023 Apr 13;3(4):e0001816. doi: 10.1371/journal.pgph.0001816 (PMC10101378; doi:10.1371/journal.pgph.0001816)
Supplement: Attachment — Submitted filename: Response to Reviewers.docx [file pgph.0001816.s003.docx]

We thank the Editor and Reviewers for considering our manuscript for potential publication in *PLOS Global Public Health*. We have revised our submission as detailed below, and hope the revised version will be found suitable for publication.

**Editor comments:**

1. *Please add additional context about your clinic and its care and cost policies/practices for undocumented migrants / non-insured.*

We have added the following text to the methods section:

“The business organization that administers the school of medicine clinics at the university has a financial assistance policy for guarantors with annual household income less than 300% of the federal poverty level (FPL) that provides adjustment off charges in the amount of 50%, 70% and 90% for guarantors with household incomes of 201% to 300% FPL, 101% to 200% of FPL and < 100% of FPL, respectively. These discounts apply to Evaluation and Management Services, Diagnostic Procedures and Treatment services that are deemed non-elective and medically necessary. The total FPL discount must not exceed 5% of a clinical department’s total charges. Uninsured patients that do not qualify for the FPL discount above are eligible for 30% adjustment off charge amount for Evaluation and Management Services, Diagnostic Procedures and Treatment services that are deemed non-elective and medically necessary outpatient care. There are no prohibitions against providing care for undocumented migrants and/or non-insured patients.” (Page 6-7, lines 130-142)

And

“Together these clinics offer treatment for many chronic diseases, both common and rare, affecting children including obesity, hypertension, kidney disease, congenital and acquired heart diseases, asthma and other pulmonary diseases, apnea and other sleep disturbances, type 1 and type 2 diabetes mellitus and other endocrine problems, chronic constipation, celiac and inflammatory bowel diseases, gastro esophageal reflux, and liver and gall bladder diseases.” (Page 7, lines 147-152)

2. *Please revise for a global public health audience, paying particular attention to US-centric and medical language throughout.*

We have revised our manuscript to make it more accessible and relevant for a global public health audience, with extensive changes in the background section to identify the geographic and US region of the study, give more context around the immigration policies, provide more explanation for medical or health-system related terms, and use of term “postal” code rather than ZIP code.

3. *Please ensure all tables and figures have sufficient information to be understood at a glance.*

We have revised the tables (including addressing R2Q4) to ensure each one is as self-contained as possible and can be understood at a glance.

4. *Please provide a visual representation of attended/non-attended visits by year and ethnicity as per Reviewer 2 suggestion.*

We have added this as a new Figure 1.

5. *In the online submission form, you indicated that "Identified study data cannot be released to protect patient confidentiality. Deidentified data are available from the authors upon request.". All PLOS journals now require all data underlying the findings described in their manuscript to be freely available to other researchers, either 1. In a public repository, 2. Within the manuscript itself, or 3. Uploaded as supplementary information. This policy applies to all data except where public deposition would breach compliance with the protocol approved by your research ethics board. If your data cannot be made publicly available for ethical or legal reasons (e.g., public availability would compromise patient privacy), please explain your reasons by return email and your exemption request will be escalated to the editor for approval. Your exemption request will be handled independently and will not hold up the peer review process, but will need to be resolved should your manuscript be accepted for publication. One of the Editorial team will then be in touch if there are any issues.*

We have obtained approval from our IRB to share the deidentified data as requested. We have provided deidentified data and code for replicating our analysis as supplementary files with the revised submission. All overt patient identifiers such as Medical Record Number have been removed, and, to further protect patient confidentiality, distance from the clinic has been rounded to the nearest multiple of 10km (with all distances <10km placed in the 10km group). Rounding the distances will prevent reidentifying patient postal codes based on their distance from the ECU hospital postal code, but will lead to minor differences in the regression model coefficients and standard errors compared to what is in the manuscript.

**Reviewer 1 comments:**

1. *The authors attempt to test the hypothesis that increasingly restrictive US immigration policies (and their enforcement) may have impacted access of Latinx pediatric subspecialty clinic patients at their centre. While they identified no significant decrease in clinic visits, there are interesting findings here.*

We thank the Reviewer for their positive evaluation of our manuscript.

2. *Also, the study team identifies that there are notable design challenges in this study, as their study focuses on a specific clinical centre, on children with chronic conditions (and established therapeutic relationships with their providers), relating to subspecialty pediatric care. Despite these limitations, the study does elucideate novel information about this specific patient population and their healthcare resource utilization, along with the comparisions drawn between Latinx and non-Latinx White patients' clinic interactions via no-shows and cancelled appointments.*

We appreciate the Reviewer’s assessment and have further clarified our findings as mentioned in our responses to Reviewer 2.

**Reviewer 2 comments:**

1. *First, the overall narrative is US-oriented. Brief explanations of terminology like "Latinx" or noting that North Carolina is a US state would ensure the writing is easier to follow for a global audience. As a minor related point, the authors describe the Latinx population as the fastest growing ethnic group in the US, but I believe this is actually people of Asian origin*.

Thank you for these suggestions. We have provided additional explanations and context for these terms as described below.

We have replaced the term “Latinx” with “Latino” throughout the manuscript. While the term Latinx has been adopted to varying degrees in academic settings in the US to be more inclusive and less gender-based, the term is not preferred generally by persons of Hispanic and Latino ethnicity. For a global audience Latino is the more appropriate term.

We have updated the sentence about the growth in the Latino population in the US with the edited text below:

“The Latino population is also one of the largest and fastest growing populations among the major US ethnic groups.” P4 lines68-69

2. *Similarly, I wonder whether a public health audience will understand all the nuances pertaining to pediatric specialty care. Broadly, the article seems to be written with clinician researchers in mind, rather than a public health audience. What is "Healthy Weight" for example? More to the point, does it matter when considering the main hypothesis of the study? Perhaps some of those details either need to have more context or to move to a supplement for clinicians?*

As described above in #1 for the editors, we have added further description of the subspecialty clinics and expanded the description of the rationale for differentiating among subspecialty clinics. Because the burden of some chronic and congenital diseases is unequal across racial and ethnic groups, racial and ethnic differences in visit attendance may be confounded by clinic specialty, as a proxy for the patient populations predominantly seen in each clinic.

3. *Third, the authors seem to assume that all Latino/Latinx families in their sample are of immigrant or mixed status. (The latter is jargon, by the way, and merits explanation in the text.) It is likely they have firsthand experience in the community or with local demographic data, and so for the authors this assumption may be reasonable. As a reader, however, it remains a major methodological question: Perhaps the results are null because the analysis focuses on a largely non-immigrant, non "mixed status" community who have no immigration-related fears.*

Thank you for pointing out this use of jargon. We have added the text below to the background section:

“There are an estimated 4.5 million children in mixed status families, in which at least one family member is a US citizen and at least one family member does not have legal immigration status. It is common, for example, for children to be US-born citizens and parents and/or other household members to be a combination of legal residents, undocumented, or naturalized citizens.” P4, lines 64-68

4. *Fourth, some of the tables may be missing helpful information, e.g., the referent group for some variables?*

We have edited Tables 2 and 3 to include reference groups for attendance at each specialty clinic in 2015.

5. *Finally, it would be nice to see a visual representation of no-shows by race/ethnicity by year. Interaction terms are notoriously difficult to interpret.*

We have added a plot of predicted probabilities by year and race/ethnicity as the new Figure 1.
